# New Respiratory Inductive Plethysmography (RIP) Method for Evaluating Ventilatory Adaptation during Mild Physical Activities

**DOI:** 10.1371/journal.pone.0151983

**Published:** 2016-03-23

**Authors:** Yann Retory, Pauline Niedzialkowski, Carole de Picciotto, Marcel Bonay, Michel Petitjean

**Affiliations:** 1 U1179 Inserm, End:icap, Laboratoire de Physiologie TITAN, Montigny-le-Bretonneux, France; 2 Université de Versailles Saint-Quentin en Yvelines, UFR des Sciences de la Santé, Montigny-le-Bretonneux, France; 3 Service de Physiologie-Explorations Fonctionnelles, Hôpital Ambroise Paré, Assistance Publique-Hôpitaux de Paris, Groupe Hospitalier Paris Ile-de-France Ouest, Boulogne-Billancourt, France; Curtin University, AUSTRALIA

## Abstract

The pneumotachometer is currently the most accepted device to measure tidal breathing, however, it requires the use of a mouthpiece and thus alteration of spontaneous ventilation is implied. Respiratory inductive plethysmography (RIP), which includes two belts, one thoracic and one abdominal, is able to determine spontaneous tidal breathing without the use of a facemask or mouthpiece, however, there are a number of as yet unresolved issues. In this study we aimed to describe and validate a new RIP method, relying on a combination of thoracic RIP and nasal pressure signals taking into account that exercise-induced body movements can easily contaminate RIP thoracic signals by generating tissue motion artifacts. A custom-made time domain algorithm that relies on the elimination of low amplitude artifacts was applied to the raw thoracic RIP signal. Determining this tidal ventilation allowed comparisons between the RIP signal and simultaneously-recorded airflow signals from a calibrated pneumotachometer (PT). We assessed 206 comparisons from 30 volunteers who were asked to breathe spontaneously at rest and during walking on the spot. Comparisons between RIP signals processed by our algorithm and PT showed highly significant correlations for tidal volume (Vt), inspiratory (Ti) and expiratory times (Te). Moreover, bias calculated using the Bland and Altman method were reasonably low for Vt and Ti (0.04 L and 0.02 s, respectively), and acceptable for Te (<0.1 s) and the intercept from regression relationships (0.01 L, 0.06 s, 0.17 s respectively). The Ti/Ttot and Vt/Ti ratios obtained with the two methods were also statistically correlated. We conclude that our methodology (filtering by our algorithm and calibrating with our calibration procedure) for thoracic RIP renders this technique sufficiently accurate to evaluate tidal ventilation variation at rest and during mild to moderate physical activity.

## Introduction

Recording of tidal breathing is essential for patients undergoing evaluation for respiratory disease and reduced exercise capacity. Conventional spirometric techniques involving pneumotachometers are currently employed worldwide in pulmonary testing laboratories to record respiratory flow and calculate tidal volumes, however, the use of a mouthpiece may alter normal respiration patterns. Respiratory inductive plethysmography (RIP) has been developed and validated to evaluate tidal ventilation during quiet breathing [1–3]. RIP relies on the measurement of the current induced by an alternating magnetic field in coils, which is a function of the surface encircled by the coil [4] namely, either ribcage or abdomen deformation within inspiration and expiration phases. The use of RIP instead of a PT may avoid bias associated with breathing through a mask or mouthpiece [5].

RIP has been successfully applied to tidal ventilation recordings associated with nasal pressure, especially at night, to detect sleep apneas and to determine their origin (central versus obstructive) [6,7]. Moreover, RIP allows the evaluation of ventilation parameters such as tidal volume (Vt), inspiratory (Ti) and expiratory (Te) times and their subsequent ratios (Ti/Ttot, Vt/Ti), which are considered as major outcome variables from the bulbar respiratory control centers. However, this method has some limitations because the RIP signal might be contaminated by miscellaneous thoracic or abdominal wall motions introducing bias in determining respiratory patterns, which reflects changes in respiratory control during sleep. Despite the fact that some studies did not appear to use any filtering technique in lean subjects [2,8], or in obese subjects [9], most filters have been developed to reduce contamination in RIP signals. Most of them have been designed in the frequency domain by applying low-pass filters, wavelet filtering procedures or empirical mode decomposition [10,11]. As the occurrence and frequency of these motion artifacts are unpredictable, these sophisticated filtering methods might introduce signal distortion by eliminating both artifacts and meaningful components of RIP signals [11]. It is possible to compare RIP signals to nasal pressure signals obtained with a nasal cannula however this is crude and insufficiently accurate to directly determine tidal ventilation parameters. However, the nasal signal is less altered by body motion, so positive peak recognition can be used to determine respiratory frequency.

RIP can be used during daily activities such as walking, so specific artifacts have to be removed from the RIP signal to obtain the most accurate estimation of tidal ventilation parameter changes. Indeed, skin and fat tissue motions induced by a heel strike or arm swing can easily contaminate the respiratory signal. The aim of this study was to develop and validate a method to facilitate the use of RIP during mild physical activities. We have designed a custom-made time domain algorithm to discriminate artifacts from respiratory signals instead of using a method relying on frequency analysis. With the intention of validating our method, we compared RIP, processed using our filtering algorithm and calibration procedure, to a pneumotachometer as a reference method at rest and in submaximal exercise conditions during which the ventilatory drive is challenged. Ventilatory parameters determined by this method were found to be acceptable.

## Material and Methods

### Ethics statement

Approval for the study was obtained from the Institutional Review Board of the French learned society for respiratory medicine “- Société de Pneumologie de Langue Française–” (n° 2015–23). All subjects provided their informed written consent, and the study was conducted in accordance with the latest release of the Declaration of Helsinki.

### Subjects

Thirty healthy volunteers (7 men and 23 women, aged 36.8±14.3 yrs.) participated in this study. None had any known respiratory or neurologic disease that would interfere with tidal ventilation or locomotory behavior.

### Recordings

**Fig 1:**
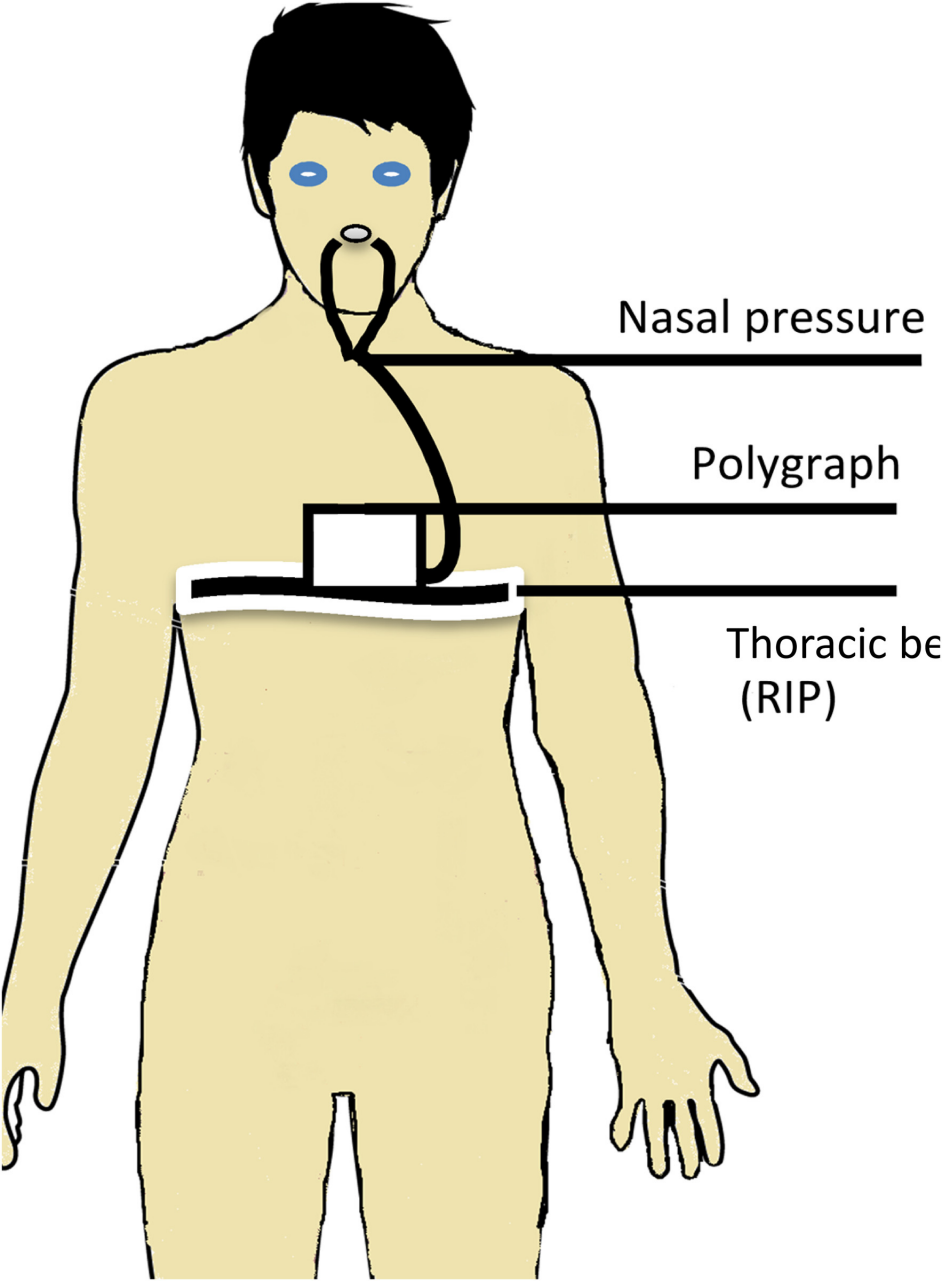
Illustration of the experimental setup including RIP thoracic belt, nasal cannula and a polygraph for acquisition of data.

Nasal pressure and RIP signals were recorded with a polygraph (NOX T3 Sleep Monitor, NoxMedical, Reykjavík, Iceland), allowing measurement of nasal pressure at a sampling frequency of 200 Hz, whereas the thoracic RIP signal was recorded at a sampling frequency of 20 Hz (Fig 1). The main ventilatory parameters (Vt, Ti, and Te) were measured by a pulmonary function test machine (Jaeger, Carefusion, Voisins-le-Bretonneux, France), including a pneumotachometer calibrated with a 3 L syringe. It allows the calculation of respiratory volumes during tidal ventilation as well as during maximal inspiratory and expiratory voluntary maneuvers. The pulmonary function test machine allowed us to measure volume changes in parallel with a change in perimeter measurements of the ribcage by RIP.

### Calibration

Prior to and after recording the ventilation activity, RIP was calibrated by simultaneously measuring volume change by PT and perimeter change by RIP while the subject remained in a standing position. The calibration consisted of simultaneously measuring ventilation at high and low levels of Vt. Once the voluntary calibration was initiated, subjects were not allowed to change their standing posture and to reach the RIP with their hands. The relationship between perimeter changes measured by RIP and volume measured by PT was determined by hypothesizing a linear regression model.

These calibration relationships were considered acceptable if their Spearman’s correlation coefficients were above 0.85. Their slope values were calculated *a posteriori* to predict volume changes from perimeter changes. Evaluation of the effect of calibration drift and coil displacement induced by activity was achieved by comparing matched slopes from the two flanking calibrations (pre- and post-).

### Protocol

Between calibration steps, recordings were performed in a quiet, warm lab room at rest and during submaximal exercise consisting of marching on the spot or knee-raising with swinging arms at different cadences. Seven bouts of one-minute measurements were made for each subject. Each measurement allowed the calculation of the median for one minute of tidal volume (Vt), inspiratory time (Ti) and expiratory time (Te).

### Data processing and description of the algorithm

Since thoracoabdominal movements and nasal airflow variation occur at the same time, we assumed that it is possible to use the nasal pressure signal as a temporal reference to identify respiratory cycles in the thoracic RIP signal. In this study, we present a time domain algorithm based on this postulate. In accordance with Liu *et al*. (2013), a second assumption is that during walking, artifact amplitude is lower than the respiratory motion itself [10]. Following the artifact removal from RIP using this algorithm, Vt was calculated by converting the voltage unit from RIP into liters in accordance with the slope of the calibration line. The difference between the end and the onset of the inspiratory phase provided the inspiratory time (Ti), whereas the difference between the end of expiratory time and the end of inspiratory time provided the expiratory time (Te). The algorithm for the treatment of RIP signals was custom-made using Matlab software (Release 2014b; the MathWorks, Inc., Natick, MA, USA).

**Fig 2:**
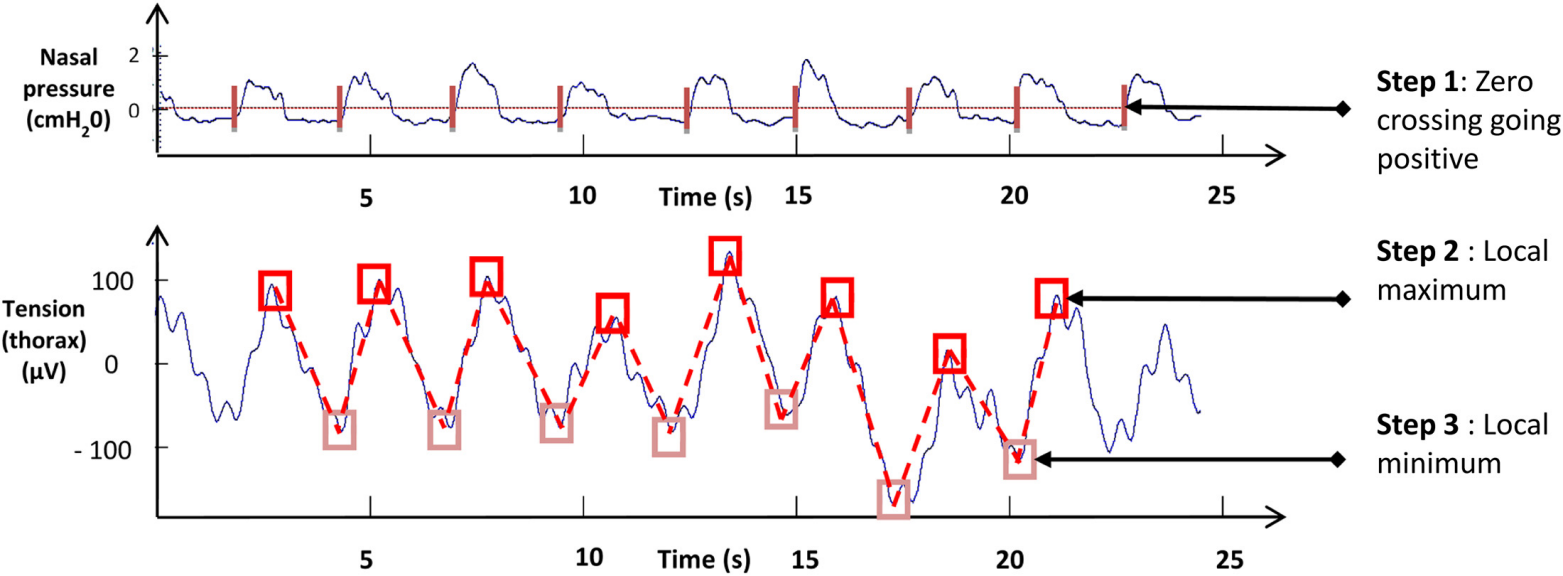
Description of the custom made algorithm in 3 steps. Step 1: recognition of the onset of the respiratory cycle by nasal signal (**vertical bars**). Step 2: searching for local RIP signal maximums (**dark squares**). Step 3: searching for local RIP signal minimums (**light squares**). Illustration of the results after treatment by the algorithm 1(**bottom panel**): treated signal (**dotted line**) superimposed on the raw signal (**continuous line**).

First step was to determine the onset of each respiratory cycle from the nasal pressure signal, characterized by a positive going zero-crossing value as:

$$i\;:\{\begin{array}{c} x_{i\;\;}\geq 0\\ x_{i-1}<0 \end{array}$$

where *x*_*i*_ is the value *x* of nasal pressure signal for time index *i* corresponding to the onset of a respiratory cycle (Fig 2).

Second step was to search for local RIP signal maximums and their indices between respiratory cycle onsets determined in step 1 as:

∀x∈[a,b], f(x) ≤f(X)

where *a* and *b* are the time indices of the RIP signal corresponding to the onset of two consecutive respiratory cycles of nasal pressure signaling. *X* is the local RIP signal maximum of a respiratory cycle (Fig 2).

Third step was to determine local minimum values and their time indices between indices found in step 2 as:

∀x∈[a,b], f(x) ≥f(X)

where *a* and *b* are time indices corresponding to two consecutives local maximum indices determined in the previous step and *X* is the local RIP signal minimum (Fig 2).

By retaining only local minimums and maximums determined in the previous steps, artifacts, which are of smaller amplitude, are not considered when calculating the ventilatory parameters.

### Statistical method

Statistical analyses were performed by using GraphPad Prism version 5.01 for Windows (GraphPad Software, San Diego California USA, www.graphpad.com). Data normality was first tested by using a D’Agostino and Pearson omnibus normality test [12]. As most of the data were not normally distributed, correlations between RIP and PT values were evaluated by the Spearman coefficient calculation for each parameter recorded (Vt, Ti, Te, Ti/Ttot and Vt/Ti). Slopes of these relationships were determined by linear regressions. Biases between RIP and PT were evaluated by the Bland and Altman method–in several places[13]. Comparison of slope values from flanking calibrations were realized with a Wilcoxon matched-pairs signed rank test.

## Results

This study evaluated the performance of RIP processed by our algorithm versus determination of ventilatory parameters by PT as a reference method. Performance of this method was evaluated for each parameter (Vt, Ti, Te, Ti/Ttot ratio, and Vt/Ti ratio) by correlating RIP data (processed by our algorithm) with PT values. Linear regression allows the evaluation of the slopes and intercepts of these relationships, whereas the Bland and Altman method revealed mean biases. Correlation coefficients, linear regressions, and bias values are summarized in Table 1.

**Table 1:** Summary of statistical analysis comparing the RIP signal processed by the custom made algorithm with PT. r: Spearman coefficient of correlation, p: p-value from Spearman coefficient determination, CI: Confidence intervals, SD: Standard deviation.

	r	p	Slope	Slope CI 95	Intercept	Intercept CI 95	Bias ± SD
**Vt (L)**	0.81	<0.0001	0.98±0.04	0.91 to 1.04	-0.02±0.04	-0.09 to 0.06	-0.04±0.24
**Ti (s)**	0.92	<0.0001	0.97±0.02	0,92 to 1.02	0.06± 03	-0,02 to 0,15	0,02±0.14
**Te (s)**	0.94	<0.0001	0.85±0.02	0,80 to 0,89	0.17± 0.04	0,09 to 0,25	-0.09± 0.18
**Ti/Ttot**	0.72	<0.0001	0.70±0.05	0,61 to 0,80	0.15±0.02	0,11 to 0,19	-0.02±0.03
**Vt/Ti (L.s**^**-1**^**)**	0.87	<0.0001	1.06±0.04	0,98 to 1,15	-0,08±0,04	-0,16 to -0,01	-0.03± 0.19

**Fig 3:**
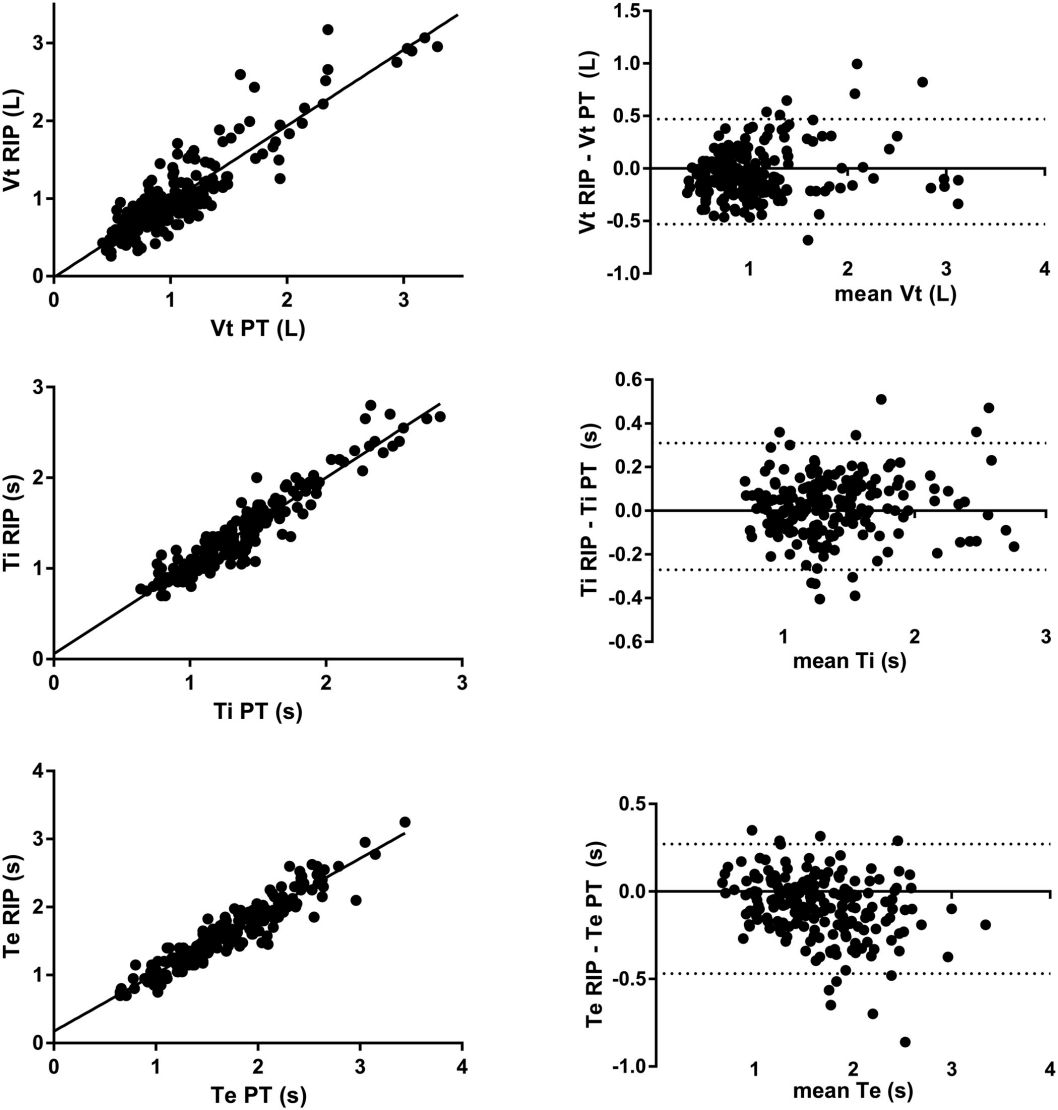
Evaluation of Vt, Ti and Te by RIP signals processed by the custom made algorithm. The linear relationship between Vt determined by PT, and Vt determined by RIP plus the algorithm is shown in the upper left panel. Bland and Altman’s analysis of Vt determined by PT, and Vt determined by RIP plus the algorithm is shown in the upper right panel with bias (long dotted line) and limit of agreements (short dotted line). The linear relationship between Ti determined by PT, and Ti determined by RIP plus the algorithm is shown in the middle left panel. Bland and Altman’s analysis of Ti by PT and by RIP plus the algorithm is shown in the middle right panel with bias (long dotted line) and limit of agreements (short dotted line). The linear relationship between Te determined by PT, and Te determined by RIP plus the algorithm (lower left panel). Bland and Altman’s analysis of Te by PT and Te by RIP signal plus the algorithm (lower right panel) with bias (long dotted line) and limit of agreements (short dotted line).

This method shows a highly significant correlation with PT for Vt values (r = 0.81; p<0.0001). The linear relationships between PT and RIP processed by the algorithm showed a slope close to 1 (0.98) with an intercept close to zero (-0.02 L) (Fig 3). Bland and Altman’s analyses revealed a bias of 0.04 L.

Ti values using our method and measured by PT showed a highly significant correlation (r = 0.92; p<0.0001) with a slope close to 1 (0.97 s) and an intercept close to 0 (0.06 s) (Fig 3). Bias from Bland and Altman analyses was negligible (0.02 s).

The performance of Te was lower compared to the performance for Ti. Indeed, even if the correlation between PT and our method was good (r = 0.94; p<0.0001), the slope and intercept values from linear regression between Te values from PT and Te values were further from the identity line compared to Ti (0.85 and 0.17 s, respectively). Likewise, the bias value for Te determination was higher (-0.09 s) than for Ti (Fig 3).

**Fig 4:**
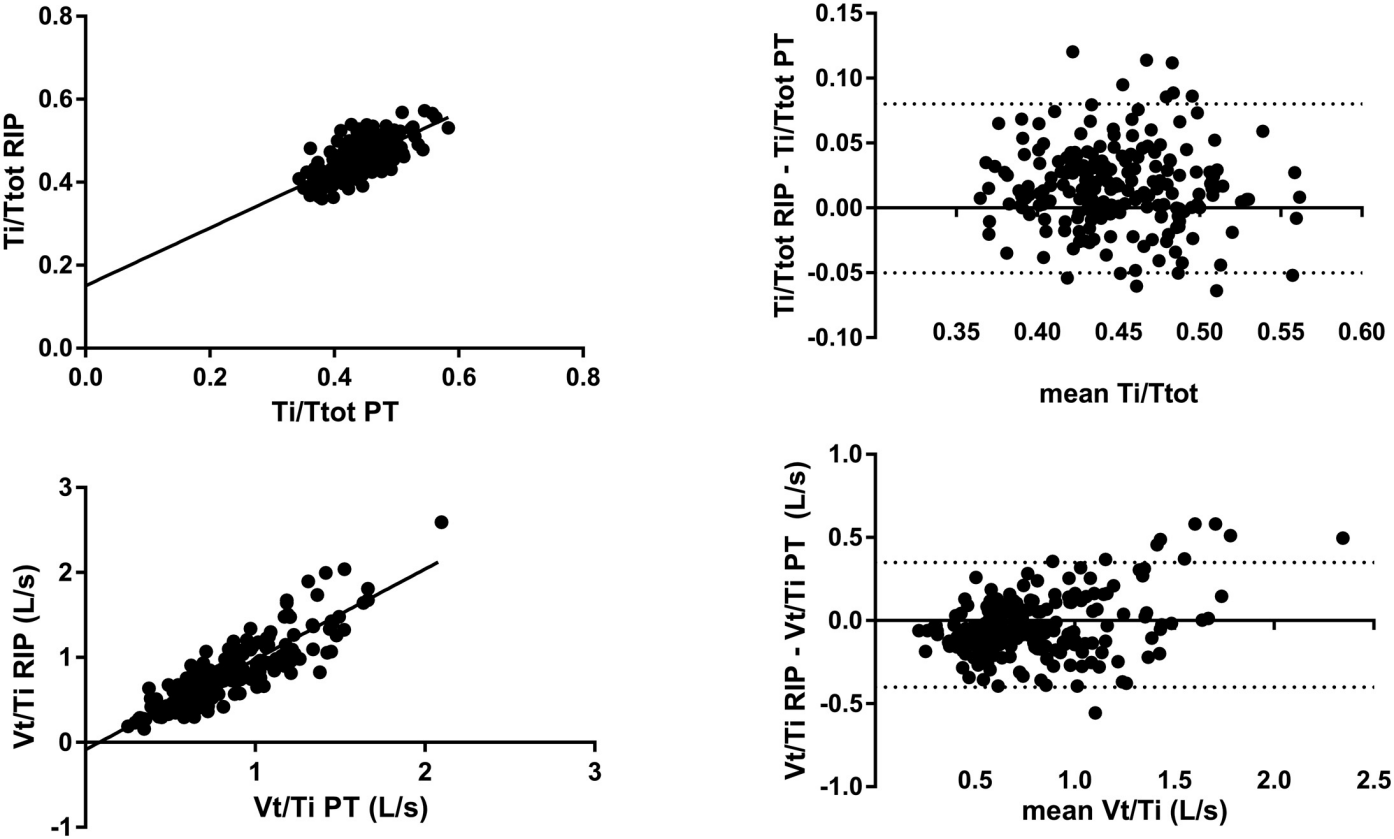
Evaluation of Ti/Ttot and Vt/Ti ratios determined by RIP signals processed by the custom made algorithm. The linear relationship between Ti/Ttot ratio by PT and the Ti/Ttot ratio by RIP signal treated by the algorithm (upper left panel). Bland and Altman’s analysis of Ti/Ttot ratio determined by PT and Ti/Ttot ratio determined by RIP signal treated by the algorithm (upper right panel) with bias (long dotted line) and limit of agreements (short dotted line). Linear relationship between Vt/Ti ratio determined by PT and the Vt/Ti ratio determined by RIP signal treated by the algorithm (lower left panel). Bland and Altman’s analysis of Vt/Ti ratio determined by PT and Vt/Ti ratio determined by RIP signal treated by the algorithm (lower right panel) with bias (long dotted line) and limit of agreements (short dotted line).

Correlation between Ti/Ttot ratio by PT and by our algorithm was highly significant but the Spearman coefficient was low (r = 0.72; p<0.0001) (Fig 4). Consequently, the slope of relationship between PT and this method was in accordance with a slope further away from 1 and an intercept of 0.16.

A comparison of Vt/Ti ratio measured by PT and by our method shows a good performance of our algorithm (Fig 4). Indeed, the results showed a highly significant correlation between PT and our method, a slope close to 1 (1.06 L/s), an intercept close to 0 (-0.08 L/s), and a bias of 0.03 L/s.

Slope values of relationships from flanking calibrations were not statistically different (p = 0.94). Moreover, the linear regression from the relationship between these slopes exhibited parameters very close to the identity line (r = 0.96, slope = 0.99 ± 0.07, and intercept = 0.00± 0.01).

## Discussion

In this study, we aimed to validate a simple time domain method to evaluate tidal ventilation using filtered thoracic RIP signal. By comparing tidal breathing parameters from filtered RIP and PT signals, we obtained good concordance and proportionality. Our results clearly demonstrated that RIP thoracic and simultaneous nasal pressure signals can be a useful tool for monitoring ventilation during mild, submaximal physical activities.

Our original RIP method differs from previous approaches in that we use only the thoracic belt associated with the nasal pressure signal. Indeed, we believe that the abdominal belt signal might be necessary for increasing accuracy and retaining two degrees of freedom, as previously described by Konno and Mead [14]. However, this also increases the probability of breaking assumptions made about linearity in the movement-volume relationship when asynchrony between the thorax and the abdomen occurs. It is now well known that asynchrony, occurring when respiratory load increases, can often produce inaccuracy and variability in determining Vt from RIP [9,15,16]. Explanations for this inaccuracy are controversial, and the use of non-linear calibration might solve this issue when using a model with two degrees of freedom [16]. However, non-linear calibration is not easy, and to our knowledge, there is no available commercial application that would allow it to be performed routinely. By using only the thoracic RIP signal, Vt determination might not be affected by the presence or absence of asynchrony during calibration or measurement causing errors [16]. We are aware of the possible lack of accuracy related to this model with only one degree of freedom, but this has the advantage of being more stable, regardless of the involvement of the abdomen, and it provides a linear relationship between the thorax perimeter change and volume change, as previously described [17]. Since we did not compare the use of one thoracic belt with a more standard thoracic and abdominal RIP device, we cannot rule out the absolute benefit of using thoracic belt alone. However, we might argue that our Vt, Ti and Te values from one thoracic belt correlate with those obtained from the pneumotachometer. Another limitation of the method concerns the higher values of Vt beyond 1.5 L, with higher scattering and less precise determination. This effect, as seen in Fig 3, was substantial and demonstrates that the RIP estimation of minute ventilation is merely adapted for submaximal exercise with a slight increase of Vt and respiratory rate. This has already been reported in previous studies, indicating that RIP cannot be used to record ventilation during an incremental exercise test up to the maximum of the subject’s respiratory capacity, because of the lack of accuracy for the highest values of Vt [4].

Average biases for the determination of respiratory time Ti and Te are low. Indeed, good determination of the beginning and end of respiratory cycles suggests obtaining an artifact-free signal. However, the intercepts from the linear regressions show a slight tendency towards overestimation. This suggests that the RIP would detect little chest movements prior to the onset of airway pressure changes. Moreover, these negligible biases indirectly support good performance of our algorithm to confidently remove artifacts. The correlation of the Ti/Ttot ratio measured by PT and determined by our method was lower compared to those obtained for Ti or for Te. The low range during exercise is unlikely to yield high correlation coefficient value. Meanwhile, Vt/Ti had higher and less scattered correlation compared to Ti/Ttot, except for the highest values. This can be explained by the fact that Vt/Ti has a flow dimension closer to airflow at the mouth compared to either Vt or Ti separately.

**Fig 5:**
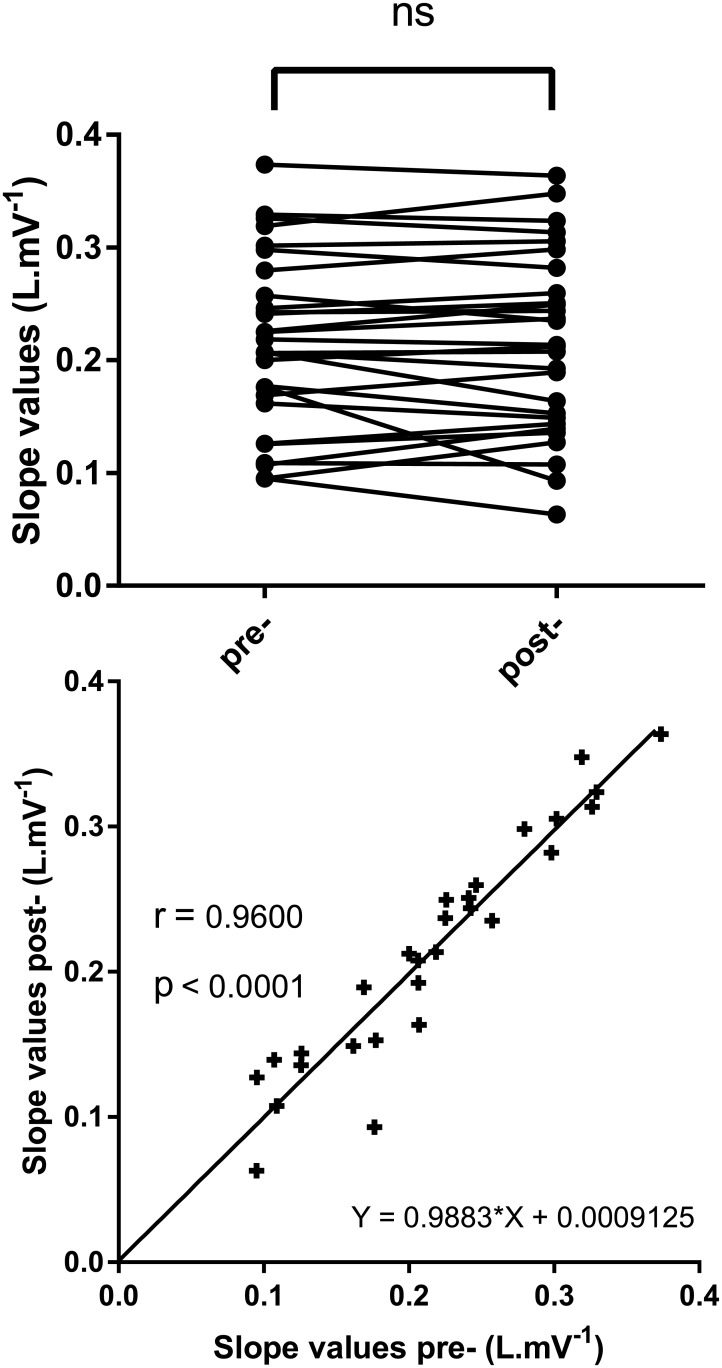
Evaluation of slopes from linear relationships between RIP and PT before and after activity. Variations in slopes (left panel) ns: not significant. Relationship between slopes before and after activity (right panel) r: Spearman correlation coefficient; p: p-value.

In this study, we chose to delimit the measurement phase by two calibrations to minimize the possible effect of calibration drift and coil displacement, which could occur with activity-induced movements between calibrations and lead to inaccuracy [9]. Thus, the slope calculated to predict Vt from the chest circumference change considers the values of the two calibration flanking measures. As shown in Fig 5 (upper panel) there was no statistical difference between the slopes values pre- and post- test. There was also a high positive correlation (r = 0.96 p<0.0001) between the individual slope values surrounding the identity line (Fig 5 bottom panel). This indicates that in most cases, the effects of calibration drift and belt displacements were negligible. Nevertheless, the flanking calibration method used here seems to be useful for at least a few cases for which the drift exists. To respect the calibrations parameters depending on the relationship between chest wall movement and volume change, subjects were not allowed to change their posture. Indeed, it has been shown that posture needs to be considered when determining the breathed volume from thoracoabdominal deformation [14].

The PT used in this study does not allow us to analyze breath-by-breath ventilation of a subject *a posteriori*. This is the reason why we considered parameters such as the median calculated over one minute for each measurement. This might have reduced the scattering of our data. However, the results from Witt *et al*. (2006) calculated by using breath-by-breath data yielded the same results [2]. This can also minimize the intra-individual scattering contribution to the variance of all measured parameters.

We aimed to validate thoracic RIP signals that contained artifacts treated by our algorithm by comparing them with PT. Since our PT did not allow us to perform measurements with body mobility, subjects had to walk on the spot raising their knees and swinging their arms to exaggerate artifact occurrence. This is not exactly walking, but as this type of body movement mimics gait, we observed tissue motion artifacts as well as an increase in Vt up to two-fold or above, which is in the range of minute ventilation recorded during walking on a treadmill [18]. Thus, this kind of submaximal exercise provides a good model of ventilation challenge and artifacts, which may be recorded during whole body movement or walking (Fig 2).

The results from our algorithm were compared with the PT as a reference method, which directly measures the airflow through the upper airways. Unlike RIP, PT is not affected, or at least is affected much less, by subcutaneous tissue motions. Therefore, our results (provided with our method) are comparable to those of the reference method which indicate efficiency and accuracy. This is particularly true for Vt, Ti and Te. Nevertheless, the results are less strong for the Ti/Ttot ratio, likely because they represent a combination of different raw data and measurement uncertainties. Thus, our method seems efficient enough to provide tidal volume and time values close to those given by integration of the airflow. Despite this performance, we have to keep in mind that our thoracic RIP device is not dedicated to absolute volume measurement but to estimation of tidal parameter changes related to moderate exercise.

Important conditions for running this algorithm need to be considered. Indeed, to determine the limits of cycles on thoracic signals, it is necessary to simultaneously record airflow variation and thoracic perimeter changes, which is not the case if subjects breathe exclusively through the mouth. Moreover, the algorithm is efficient in reducing low amplitude artifacts but is unable to parse extreme values over the range of tidal cycle mean amplitudes. According to the results of the present study, the algorithm seems appropriate for usual artifact removal when avoiding high amplitude artifacts (with a close-fitting belt) and recording the nasal pressure signal (exclusion of nasal obstruction).

Our results suggest that thoracic RIP alone could be used in routine practice with appropriate time domain filtering for artifact removal and appropriate calibration, which considers mild displacement of belts. We conclude that with this method allows the pertinent evaluation of tidal ventilation (during a 6-minute walk test for example), without a PT thus without alteration of spontaneous ventilation. There are still requirements to test our time domain approach to RIP measurement with subjects of high body mass index, and during periods of natural physical activity.
